# Validity and reliability of the Finnish version of the Multiple Sclerosis Impact Scale‐29

**DOI:** 10.1002/brb3.725

**Published:** 2017-05-17

**Authors:** Eija Rosti‐Otajärvi, Päivi Hämäläinen, Anna Wiksten, Tanja Hakkarainen, Juhani Ruutiainen

**Affiliations:** ^1^ Department of Neurosciences and Rehabilitation Tampere University Hospital Tampere Finland; ^2^ Finnish Neuro Society Masku Finland; ^3^ StatFinn Oy Turku Finland; ^4^ Novartis Finland Oy Espoo Finland; ^5^ University of Turku Turku Finland

**Keywords:** health‐related quality of life questionnaire, multiple sclerosis, Multiple Sclerosis Impact Scale, reliability, self‐report, validity

## Abstract

**Background:**

The Multiple Sclerosis Impact Scale‐29 (MSIS‐29) has been increasingly used to evaluate the self‐perceived impact of multiple sclerosis (MS) on a patient.

**Objectives:**

The aim of this study was to evaluate the psychometric properties of the Finnish version of MSIS‐29 in patients with MS.

**Methods:**

A total of 553 patients with MS completed the MSIS‐29 and self‐administered questionnaires capturing information on demographics, disease characteristics and severity, perceived quality of life (EuroQol 5D‐3L instrument), and fatigue (Fatigue Severity Scale).

**Results:**

The data quality for MSIS‐29 was excellent, with 99.5% computable scores for the MSIS‐29 physical scale and 99.3% for the MSIS‐29 psychological scale. Floor and ceiling effects were minimal. Excellent Cronbach's alpha values of 0.97 and 0.90 were seen for MSIS‐29 physical and psychological subscales, respectively. The physical subscale showed highest correlations with measures of physical functioning, such as disease severity and the mobility domain of the quality of life. Similarly, the psychological subscale showed highest correlations with self‐reported fatigue and the anxiety/depression domains of the quality of life. MSIS‐29 physical scores related strongly to disease severity, whereas the MSIS‐29 psychological scores increased in mild disease but declined in more severe disease forms.

**Conclusion:**

The Finnish version of MSIS‐29 has satisfactory psychometric properties. Consistent with the previous recommendations, the use of two MSIS‐29 subscale scores instead of a total score was supported.

## 1 INTRODUCTION

Multiple sclerosis (MS) is an incurable progressive neurological disease, with a wide spectrum of symptoms covering fatigue, visual disturbances, limb weaknesses, deficits in coordination and balance, bladder and bowel disturbance, pain, reduced heat tolerance, dysarthria, cognitive dysfunction, and depression (McDonald & Ron, 1999). The disease thus has significant social, psychological, and physical impacts on the patient and thereby also affects the perceived quality of life. In the fast‐growing field of immunotherapeutic treatments, there is an increasing need for sensitive and clinically relevant assessment methods to describe the effects of MS on patients. Psychometrically robust methods to evaluate physical and psychological disease burden of MS are also needed in population‐based and rehabilitation studies as well as for the continuous evaluation of individual patients.

Several measurement scales, both disease specific and generic, have been used to evaluate functioning and quality of life of patients with MS. Clinician outcomes, that primarily capture functional disability include Expanded Disability Status Scale (EDSS) (Kurtzke, 1983) and Multiple Sclerosis Functional Composite (MSFC) score (Cutter et al., 1999). Patient‐reported outcomes, that can capture broader effects on patient's quality of life, include Functional Assessment of Multiple Sclerosis (FAMS) (Cella et al., 1996), Multiple Sclerosis Quality of Life (MSQOL‐54) (Vickrey, Hays, Harooni, Myers, & Ellison, 1995), and the Medical Outcomes 36‐item Short‐Form Health Survey (Hobart, Freeman, Lamping, Fitzpatrick, & Thompson, 2001). For decades, outcome measurement in MS has relied particularly on EDSS. While EDSS is the most widely used assessment tool to capture the level of physical disability, it has a poor ability to take into account other aspects, such as fatigue and cognition. While the generic patient‐reported outcome measures, such as quality of life or perceived health or disability questionnaires, enable comparisons across diseases and with normative population, they do not recognize MS‐specific symptoms. Furthermore, none of the aforementioned MS‐specific questionnaires have been developed using the standard psychometric approach of reducing a large item pool generated de novo from the people with MS.

The Multiple Sclerosis Impact Scale‐29 (MSIS‐29) was developed in 2001, and since then has been used increasingly in both research and clinical settings (Hobart, Lamping, Fitzpatrick, Riazi, & Thompson, 2001). MSIS‐29 is a measure of the perceived physical and psychological impact of MS from the patient's perspective. It was developed as a MS‐specific scale using a standardized psychometric approach of reducing an item pool generated from patient interviews, expert opinion, and literature review (Hobart et al., 2001). It is a questionnaire structured in two subscales — a 20‐item scale for the physical impact and a 9‐item scale for the psychological impact of the disease. The items are answered in a five‐point Likert scale ranging from one (“not at all”) to five (“extremely”) (Table 1). The two subscale scores are generated by summing individual items and then transformed to a 0–100 scale. Higher scores indicate a more severe disease burden. The total score can be reported, but is not recommended (Hobart et al., 2001). MSIS‐29 has been suggested for use in cross‐sectional studies to describe the impact of MS, in longitudinal studies to monitor the natural history of the disorder, and in clinical trials to evaluate therapeutic effectiveness from the patients’ perspective (Hobart et al., 2001).

**Table 1:** The Multiple Sclerosis Impact Scale‐29: English and Finnish versions

In the past 2 weeks, how much has your MS limited your ability to:/Miten paljon MS‐tauti on viimeisen kahden viikon aikana rajoittanut kykyäsi:
1. Do physically demanding tasks?/Suoriutua ruumiillisesti raskaista tehtävistä?
2. Grip things tightly (e.g. turning on taps)?/Ottaa kädellä tiukka ote (esim. vääntää hana auki)?
3. Carry things?/Kantaa tavaroita käsin?
In the past 2 weeks, how much have you been bothered by:/Miten paljon viimeisen kahden viikon aikana seuraavat asiat ovat haitanneet sinua?
4. Problems with your balance?/Tasapaino‐ongelmat?
5. Difficulties moving about indoors?/Liikkumisvaikeudet sisätiloissa?
6. Being clumsy?/Kömpelyys?
7. Stiffness?/Jäykkyys?
8. Heavy arms and/or legs?/Kädet ja/tai jalat tuntuvat painavilta?
9. Tremor of your arms or legs?/Käsien tai jalkojen vapina?
10. Spasms in your limbs?/Lihaskrampit käsissä tai jaloissa?
11. Your body not doing what you want it to do?/Kehosi ei toimi tahtosi mukaan?
12. Having to depend on others to do things for you?/Riippuvaisuus muiden avusta?
13. Limitations in your social and leisure activities at home?/Sosiaalisen kanssakäymisen ja vapaa‐ajan vieton rajoitteet kotona?
14. Being stuck at home more than you would like to be?/Kotiin juuttuminen, enemmän kuin olisit halunnut?
15. Difficulties using your hands in everyday tasks?/Vaikeudet käyttää käsiä arkiaskareissa?
16. Having to cut down the amount of time you spent on work or other daily activities?/Pakko rajoittaa työhön tai muihin päivittäisiin aktiviteetteihin käytettyä aikaa?
17. Problems using transport (e.g. car, bus, train, taxi etc.)?/Vaikeudet käyttää liikennevälineitä (esim. oma auto, taksi, linja‐auto)?
18. Taking longer to do things?/Asioiden tekeminen vie enemmän aikaa?
19. Difficulty doing things spontaneously (e.g. going out on the spur of the moment)?/Vaikeudet tehdä asioita hetken mielijohteesta (esimerkiksi lähteä ulos)?
20. Needing to go to the toilet urgently?/Tarve päästä kiireesti WC:hen?
21. Feeling unwell?/Huonovointisuus?
22. Problems sleeping?/Univaikeudet?
23. Feeling mentally fatigued?/Henkinen uupumus?
24. Worries related to your MS?/Huolestuneisuus MS‐taudista?
25. Feeling anxious or tense?/Ahdistuneisuuden tunne tai jännittyneisyys?
26. Feeling irritable, impatient, or short tempered?/Ärtyneisyys, kärsimättömyys tai lyhytjännitteisyys?
27. Problems concentrating?/Keskittymisvaikeudet?
28. Lack of confidence?/Luottamuksen puute (omaan itseen, omiin mahdollisuuksiin)?
29. Feeling of depressed?/Masennus?

Validation studies of MSIS‐29 have been conducted in addition to the original English version, per our knowledge, for the Norwegian (Smedal, Johansen, Myhr, & Strand, 2010), Korean (Huh et al., 2014), German (Schäffler et al., 2013), Dutch (Hoogervorst, Zwemmer, Jelles, Polman, & Uitdehaag, 2004), Polish (Jamroz‐Wisniewska et al., 2007), and Persian (Ayatollahi, Nafissi, Eshraghian, Kaviani, & Tarazi, 2007) versions. Additionally, preliminary findings have supported the stability of MSIS‐29 across eight European countries (Hobart et al., 2004). Acceptable psychometric properties of MSIS‐29 in community‐ and hospital‐based samples have also been reported. Convergent validity of MSIS‐29 has been supported by high correlations between MSIS physical score and measures of physical functioning, such as disability scales (e.g., EDSS and MSFC) (Hoogervorst et al., 2004; Huh et al., 2014; McGuigan & Hutchinson, 2004; Schäffler et al., 2013), self‐reported health status (McGuigan & Hutchinson, 2004; Smedal et al., 2010), as well as the physical domains of quality of life questionnaires (Hobart et al., 2001; Huh et al., 2014; Learmonth, Hubbard, McAuley, & Motl, 2014; Riazi, Hobart, Lampling, Fitzpatrick, & Thompson, 2002). Similarly, high correlations have been found between MSIS‐29 psychological scores and depressive symptoms (McGuigan & Hutchinson, 2004) and the psychological domains of quality of life questionnaires (Hobart et al., 2001; Huh et al., 2014; Learmonth et al., 2014; Riazi et al., 2002). MSIS‐29 has shown high internal consistency, as analyzed using Cronbach's alpha (Gray, McDonnell, & Hawkins, 2009; Hobart et al., 2001; Huh et al., 2014; Jones et al., 2013; McGuigan & Hutchinson, 2004; Riazi et al., 2002; Smedal et al., 2010), as well as a high test‐retest reliability (Learmonth et al., 2014; Smedal et al., 2010). The MSIS‐29 physical scale has been found responsive to change in EDSS (McGuigan & Hutchinson, 2004), steroid therapy, and rehabilitation (Hobart, Riazi, Lampling, Fitzpatrick, & Thompson, 2005). Furthermore, MSIS‐29 has been found to be a reliable and valid instrument when used by proxies (van der Linden et al., 2005) and via the internet (Jones et al., 2013).

The purpose of this study was to evaluate the psychometric properties of the Finnish version of MSIS‐29 in terms of data quality, scaling assumptions, acceptability, validity, and reliability in a large sample of MS patients including all clinical phenotypes, and with a wide range of disability. Additionally, the dimensional structure of MSIS‐29 was evaluated.

## 2 MATERIALS AND METHODS

### 2.1 Patients

This was a retrospective, cross‐sectional mail survey. The study protocol was approved by the ethics committee of the Hospital District of South‐Western Finland and all participants provided written informed consent. The study population included patients registered with the Finnish Neuro Society, a national patient association in Finland. The inclusion criteria comprised diagnosis of MS, age ≥ 18 years, a membership in the Finnish Neuro Society for at least 1 year, a permission to receive mail from the association, ability to complete the survey in the Finnish language, no illness other than MS that could limit their participation, and not enrolled in any other clinical trial.

### 2.2 Outcome measures

The study population and methods have been described in detail previously (Ruutiainen, Viita, Hahl, Sundell, & Nissinen, 2016). Briefly, the patients were required to complete the survey questionnaire or were interviewed via telephone using the Finnish questionnaire adapted from previous, multinational studies (Karampampa, Gustavsson, & Miltenburger, 2013). The questionnaire included demographic background variables and disease information (e.g., year of diagnosis, type of MS, and self‐assessment of disease severity by Patient Assessment of Expanded Disability Status Scale (EDSS) Levels, a method widely used in cost‐of‐illness studies in MS (Kobelt, Berg, Lindgren, & Jönsson, 2006). The perceived quality of life was evaluated using the generic EuroQol 5D‐3L instrument (EQ‐5D) including five domains of well‐being (mobility, personal care, usual activities, pain/discomfort, and anxiety/depression) using a social tariff established with the general population in UK (Euroqol Group, 1990). The visual analogue scale (VAS) was used to assess patients’ perceived health state on a scale of 0 (worst imaging health state) to 100 (best imaginable health state) (Euroqol Group, 1990). The perceived severity of fatigue was evaluated using the Fatigue Severity Scale (FSS) (Krupp, Larocca, Muir‐Nash, & Steinberg, 1989). In addition, the physical and psychological impacts of the disease were assessed using MSIS‐29 (Hobart et al., 2001).

The translation of MSIS‐29 from English into Finnish was accomplished by a clinician familiar with MS. The Finnish version was then back‐translated to English by a professional translator. The Neurological Outcome Measures Unit of the Institute of Neurology conducted a similarity check of the original and back‐translated version (Hobart et al., 2004). The items of MSIS‐29 are presented in English and Finnish in Table 1.

### 2.3 Statistical methods

The following psychometric properties of MSIS‐29 were evaluated using standard methods (Nunnally & Bernstein, 1994):

- Data quality: The percentage missing data and percentage computable scores were determined.
- Scaling assumptions: Item mean scores and standard deviations (*SD*), skewness, and item to total correlations were determined.
- Acceptability: Score range, mean scores, floor/ceiling effects, and skewness were estimated.
- Reliability: Cronbach's alpha with 95% confidence intervals (CIs) and Cronbach's alpha when one item is deleted were calculated.
- Validity: *Internal* validity was evaluated by examining the intercorrelation between the MSIS‐29 physical and psychological scores. For the evaluation of *convergent* and *divergent* validity of MSIS‐29, Spearman correlation coefficients were used to examine the relationship between the MSIS‐29 physical and psychological scores with the disease severity (Patient Assessment of EDSS Levels), quality of life (EQ‐5D/utility, mobility, anxiety/depression, and VAS), and fatigue (FSS). *Known‐group* validity was determined by examining MSIS‐29 scores for subgroups of patients. We predicted that (i) patients who were retired due to their MS would have higher scores (i.e., poorer health) than those who were still employed; (ii) patients with greater disease severity would have higher scores than those with milder disease severity; (iii) patients with progressive disease phenotype (secondary or primary progressive) would have higher scores than those with relapsing‐remitting form of the disease; (iv) older patients would have higher scores than would younger patients; and (v) patients of different gender would have similar scores. Student's *t*‐tests were used when comparing two groups (gender and employment status), while for comparing three or more groups (disease phenotype, severity of the disease, and age groups), analyses of variance (ANOVA) were used. The Tukey's honest significance difference test was used for post hoc pairwise comparisons following ANOVAs.
- Unidimensionality: A confirmatory factor analysis (CFA) was used to evaluate the dimensional structure of MSIS‐29. Comparative fit index (CFI) was calculated to evaluate the fit of MSIS‐29 physical, psychological, and total scores in a unidimensional model.

## 3 RESULTS

### 3.1 Demographic and clinical characteristics of the sample

Overall, 553 patients completed the questionnaire and were included in the analysis. The study sample was representative of all ages, MS phenotypes, and levels of disability (Table 2).

**Table 2:** Sample demographics and disease characteristics (*n *=* *553)

Gender, *n* (%)	
Female	435 (78.7)
Age, years	
Mean (*SD*)	53.8 (11.4)
Range	21–88
Current employment situation, *n* (%)	
Employed or self‐employed	195 (35.3)
Student	2 (0.4)
Unemployed	23 (4.2)
On disability pension (any reason)	223 (40.3)
On retirement pension	110 (19.9)
Diagnosis	
Age at diagnosis, mean (*SD*)	37.4 (10.1)
Years since dg, mean (*SD*)	16.4 (9.3)
Disease phenotype, *n* (%)	
Relapsing‐remitting	244 (44.1)
Primary progressive	94 (17.0)
Secondary progressive	160 (28.9)
Unknown	55 (10.0)
Disease severity (EDDS score)	
Mean (*SD*)	4.0 (2.5)
Range	0 – 9

EDDS, Patient Assessment of Expanded Disability Status Levels; *SD*, standard deviation.

### 3.2 Data quality

The percentage of missing data for items was low (1.3%), and the percentage of computable scale scores was high for both MSIS‐29 physical (99.5%) and psychological (99.3%) scales (Table 3).

**Table 3:** Data quality, scaling assumptions, acceptability, and reliability of the MSIS‐29

Psychometric property	MSIS‐29 physical	MSIS‐29 psychological
Data quality (*n* = 553)		
Subjects with missing items, *n* (%)	3 (0.5)	4 (0.7)
Number of missing items, *n* (%)	3 (<0.01)	5 (<0.01)
Computable scale scores, *n* (%)	550 (99.5)	549 (99.3)
Scaling assumptions (*n* = 546)		
Item mean score, range	1.7 – 3.0	1.6 – 2.2
Item *SD*, range	1.0 – 1.4	0.9 – 1.2
Item skewness, range	0.090 – 1.640	0.770 – 1.620
Item total correlation, range	0.519 – 0.857	0.472 – 0.807
Acceptability		
Possible score range	0–100	0–100
Observed score range	0–98.8	0–91.7
Score, mean (*SD*)	33.9 (24.8)	24.0 (19.8)
Floor, *n* (%)	19 (3.5)	42 (7.7)
Ceiling, *n* (%)	0 (0)	0 (0)
Skewness	0.48	0.99
Reliability		
Cronbach's alpha	0.965	0.895
95% CI	0.960 – 0.969	0.882 – 0.908
Cronbach's alpha when one item deleted: range	0.962 – 0.965	0.873 – 0.899

CI, confidence interval; MSIS‐29, Multiple Sclerosis Impact Scale; *SD*, standard deviation.

### 3.3 Scaling assumptions

Frequency distributions for item response were relatively symmetrical. For the MSIS‐29 physical, skewness was, however, outside the skewness range of −1 to +1 for item numbers 9, 13, 15, and 17, which were 1.640, 1.020, 1.010, and 1.210, respectively. Similarly, for the MSIS‐29 psychological, skewness was outside the skewness range of −1 to +1 for item numbers 21, 24, 25, and 29, which were 1.510, 1.090, 1.290, and 1.620, respectively. Items within each scale had similar mean scores and SDs. Item mean scores ranged from 1.7 to 3.0 (*SD* 1.0–1.4) for the MSIS‐29 physical scale and from 1.6 to 2.2 (*SD* 0.9–1.2) for the psychological scale. Item to total correlations were satisfactory (range: MSIS‐29 physical scale, 0.519–0.857; psychological scale, 0.472–0.807; Table 3).

### 3.4 Acceptability

The scores ranged from 0 to 98.8 for the MSIS‐29 physical scale and from 0 to 91.7 for the psychological scale. The mean (*SD*) scores were lower than the scale mid‐points (MSIS‐29 physical mean 33.9 [24.8], psychological mean 24.0 [19.8]). Floor effects were low (MSIS‐29 physical, 3.5%; psychological, 7.7%) and there were no ceiling effects to either of the subscales. Mean scale scores were not notably skewed (Table 3).

### 3.5 Reliability

The Cronbach's alpha reliability coefficient was 0.965 for the MSIS‐29 physical scale and 0.895 for the psychological scale showing a high degree of internal consistency of the scales. Deletion of a single item of MSIS‐29 did not change the Cronbach's alpha values markedly (range: MSIS‐29 physical scale, 0.962–0.965; psychological scale, 0.873–0.899; Table 3).

### 3.6 Validity

The correlations between the subscales as well as MSIS‐29 and other outcomes are summarized in Table 4. The physical scale correlated strongly with the psychological scale. The correlations of MSIS‐29 physical scale with the disease severity, quality of life (EQ‐5D/utility and mobility, and VAS) as well as fatigue were very high. Higher MSIS‐29 physical scores were associated with higher disease severity, lower quality of life, and higher fatigue. However, the correlations of the MSIS‐29 physical scale with the anxiety/depression domain in the EQ‐5D were weak. Similarly, the correlations of the MSIS‐29 psychological scale with quality of life (EQ‐5D/utility and anxiety/depression and, VAS) and fatigue (FSS) were strong. Higher MSIS‐29 psychological scores were associated with lower quality of life and higher fatigue. In contrast, the correlations between the MSIS‐29 psychological scale and the mobility domain in the EQ‐5D as well as disease severity were weak. This indicates that MSIS‐29 has adequate convergent and divergent validity.

**Table 4:** Spearman correlations to assess construct validity of the MSIS‐29 (*n *=* *553)

Construct	MSIS‐29 physical	MSIS‐29 psychological
Internal validity		
MSIS‐29 psychological	0.584	
Convergent validity		
Severity of the disease (EDDS)	0.799	
Quality of life (EQ‐5D, utility)	−0.788	−0.517
Quality of life (EQ‐5D, mobility)	0.703	
Quality of life (EQ‐5D, anxiety/depression)		0.566
Quality of life (VAS score)	−0.701	−0.487
Fatigue (FSS)	0.609	0.636
Divergent validity		
Quality of life (EQ‐5D, anxiety/depression)	0.261	
Quality of life (EQ‐5D, mobility)		0.332
Severity of the disease (EDDS)		0.275

EDDS, Patient Assessment of Expanded Disability Status Scale Levels; EQ‐5D, EuroQol 5D‐3L instrument; FSS, Fatigue Severity Scale; MSIS‐29, Multiple Sclerosis Impact Scale; VAS, visual analogue scale.

The MSIS‐29 scores also supported known‐group validity (Table 5). As predicted, mean MSIS‐29 were significantly higher for patients who were retired due to their MS than for those who were employed, when limiting the comparison to groups with patients aged <63 years (working age population). Additionally, mean MSIS‐29 were higher for patients with higher disease severity than for those with mild disease severity. Similarly, mean MSIS‐29 scores for patients with progressive disease phenotypes (secondary or primary progressive) were higher than for those with relapsing‐remitting form of the disease. In addition, mean MSIS‐29 physical scores were higher for older patients than for younger patients. Mean MSIS‐29 psychological scores were slightly higher for older patients, but the difference did not reach statistical significance. The MSIS‐29 psychological score did not differ between men and women, but men reported higher MSIS‐29 physical scores than women.

**Table 5:** MSIS‐29 group differences

Variable	MSIS‐29 physical, mean (*SD*)	MSIS‐29 psychological, mean (*SD*)
Age, years		
<40^1^	19.8 (20.8)	20.7 (20.6)
40–49^2^	29.4 (22.7)	24.0 (19.3)
50–59^3^	32.9 (23.6)	22.8 (19.8)
60–69^4^	43.2 (25.7)	25.2 (19.1)
≥70^5^	46.3 (23.2)	30.7 (21.4)
Mean difference (*F*‐test *p*‐value)	<.0001^1≠2,3,4,5; 2≠4,5; 3≠4,5^	.0877
Gender		
Female	32.7 (24.5)	24.3 (19.8)
Male	38.4 (25.4)	22.9 (19.9)
Mean difference (*t*‐test *p*‐value)	.0260	.4928
Employment status (All subjects)		
Employed or self‐employed^1^	18.3 (18.2)	17.3 (16.8)
Student^2^	10.0 (3.5)	18.1 (13.8)
Unemployed^3^	21.1 (22.8)	26.7 (18.9)
On disability pension^4^	44.3 (22.1)	28.8 (21.2)
On retirement pension^5^	43.9 (24.9)	25.8 (19.2)
Mean difference (*F*‐test *p*‐value)	<.0001^1,3≠4,5^	<.0001^1≠4,5^
Employment status (subjects aged <63 years)		
Disability pension due to MS (subjects aged <63 years)	43.3 (21.5)	28.3 (21.2)
All other subjects aged <63 years	18.8 (18.6)	18.7 (17.2)
Mean difference (*t*‐test *p*‐value)	<.0001	<.0001
EDDS		
0–3^1^	15.5 (14.4)	18.2 (16.4)
4–6.5^2^	43.6 (18.4)	29.6 (20.9)
7–9^3^	62.7 (22.6)	25.7 (21.7)
Mean difference (*F*‐test *p*‐value)	<.0001^1≠2,3; 2≠3^	<.0001^1≠2,3^
Disease phenotype		
Relapsing‐remitting^1^	21.3 (18.5)	20.3 (18.0)
Secondary progressive^2^	46.8 (20.9)	29.6 (21.1)
Primary progressive^3^	52.1 (24.6)	27.1 (20.7)
Unknown^4^	21.7 (21.5)	18.6 (17.6)
Mean difference (*F*‐test *p*‐value)	<.0001^1≠2,3; 2≠3,4; 3≠4^	<.0001^1≠2,3; 4≠2,3^

EDDS, Patient Assessment of Expanded Disability Status Scale Levels; MS, multiple sclerosis; MSIS‐29, Multiple Sclerosis Impact Scale; *SD*, standard deviation.

Superscript numbers refers to subgroups and differences between them.

**Figure 1:**
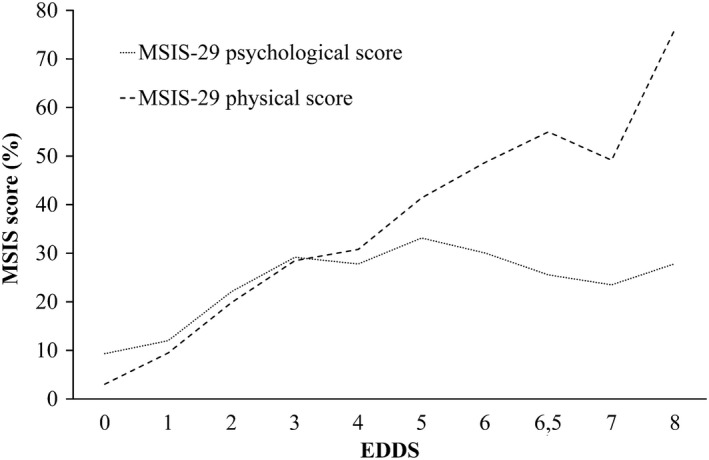
Relationship between EDDS and MSIS‐29 subscales

Further, when the relationship between the EDDS and MSIS‐29 subscales were evaluated more specifically, it was found that the MSIS‐29 physical scores related strongly to disease severity but the MSIS‐29 psychological scores increased synchronously with the EDDS score only in mild disease, peaked at EDDS 5, and declined thereafter. In case of most severe disability (EDDS 8–9) the MSIS psychological score was as high as that in EDDS 4 (Figure 1).

### 3.7 Dimensionality

The CFA results did not support the unidimensionality of the MSIS‐29. CFI for MSIS‐29 total was 0.7394 (χ^2^ = 3640.4514, *df* = 377), for the physical, subscale was 0.8829 (χ^2^ = 343.9252, *df* = 27), and for the psychological, subscale was 0.8906 (χ^2^ = 1180.0803, *df* = 170).

## 4 DISCUSSION

The aim of this study was to evaluate the psychometric properties of the Finnish version of MSIS‐29 using standard techniques evaluating data quality, scaling assumptions, acceptability, reliability, and validity. Psychometric properties were satisfactory for most of the criteria.

The data quality was excellent, with over 99% computable scale scores for both the physical and psychological subscales. Good data quality may at least partly be explained by the possibility to fill in the questionnaires via telephone interview. Scaling assumptions and acceptability were mainly good, although four items in both subscales were slightly skewed, and scale scores did not span the entire scale range. The mean (*SD*) scores remained lower than the scale mid‐points (MSIS‐29: physical 33.9 [24.8]; psychological 24.0 [19.8]). Previous studies have reported quite similar MSIS‐29 scores when the patients’ disease severity was relatively mild (Hoogervorst et al., 2004; McGuigan & Hutchinson, 2004; Smedal et al., 2010). Consistent with the findings from the validation studies in other language versions (Gray et al., 2009; Hobart et al., 2001; Jones et al., 2013; Schäffler et al., 2013), floor and ceiling effects were minimal in the present sample (no ceiling effects; floor effects for MSIS‐29 physical, 3.5%; psychological, 7.7%).

The reliability analyses included the estimation of item to total correlations and internal consistency. Item to total correlations were satisfactory for both subscales (*r*, range; MSIS‐29 physical, 0.519–0.857; psychological, 0.472–0.807) and provide evidence of item homogeneity for MSIS‐29. In the present sample, the Finnish version of MSIS‐29 showed an excellent Cronbach's alpha for both MSIS‐physical (0.97) and MSIS‐psychological (0.90) scales. Cronbach's alpha values did not significantly differ when deleting step by step one item from the physical (range 0.962–0.965) scale or psychological (range 0.873–0.899) scale. Our findings are in line with the previously reported high Cronbach's alpha values for MSIS‐29, which have varied from 0.88 to 0.97 for the physical scale and from 0.85 to 0.96 for the psychological scale (Gray et al., 2009; Hobart et al., 2001; Huh et al., 2014; Jones et al., 2013; McGuigan & Hutchinson, 2004; Riazi et al., 2002; Smedal et al., 2010).

In this study, MSIS‐29 physical subscale correlated strongly with the MSIS‐29 psychological scale (*r *=* *.58) corresponding to the previous findings where the correlations between subscales have varied from 0.44 to 0.67 (Hobart et al., 2001; Jones et al., 2013; Learmonth et al., 2014; McGuigan & Hutchinson, 2004; Ramp, Khan, Misajon, & Pallant, 2009; Riazi et al., 2002; Smedal et al., 2010). This indicates that the two scales evaluate related but distinct constructs.

MSIS‐29 showed adequate convergent and divergent validity. The MSIS‐29 physical scale correlated most strongly with disease severity (*r *=* *.80), followed by overall (*r *=* *.70‐.79) and the mobility domain of quality of life (*r *=* *.70), and fatigue (*r *=* *.61). In contrast, the correlation was weak with the anxiety/depression domain of quality life (*r *=* *.26). Accordingly, the MSIS‐29 psychological scale correlated most strongly with fatigue (*r *=* *.64), followed by anxiety/depression domain (*r *=* *.57) and overall quality of life (*r* = .49–.52), while correlation was weak with disease severity (*r *=* *.28) and moderate with the mobility domain of quality of life (*r *=* *.33). Progressive disease (higher disability and progressive phenotype) as well as retirement due to MS were found to be associated with higher MSIS‐29 scores. Interestingly, MSIS‐29 psychological scores increased with the EDDS scores in mild disease but declined in more severe disease forms. In a previous study by Gray et al. (2009), MSIS‐29 physical scores were also found to increase with disease duration, but psychological scores were significantly lower in patients with symptoms for more than 40 years. The decrease in the self‐assessed psychological burden in patients with most advanced disease may refer to adjustment to the disability. In contrast, it may also imply that the psychological subscale is insensitive to change. Older patients reported significantly higher physical but not psychological subscale scores than did younger patients. The association to age may at least partly be explained especially by increased physical disease burden along with age. As predicted, MSIS‐29 psychological scores were not affected by gender differences; instead, against our predictions and in contrast to previous findings (Hobart et al., 2001; Huh et al., 2014; van der Linden et al., 2005; Ramp et al., 2009; Riazi et al., 2002), men reported higher MSIS‐29 physical scores than women. In line with the present findings, men with MS may have a poorer prognosis than would women with MS (Vasconcelos et al., 2016) and have been found to be less physically active than were women (Anens, Emtner, Zerrerberg, & Hellström, 2014). The direction, magnitude, and pattern of correlations were, however, generally consistent with previous findings (Hobart et al., 2001; Hoogervorst et al., 2004; Learmonth et al., 2014; McGuigan & Hutchinson, 2004; Riazi et al., 2002).

Using factor analyses, two underlying subscales of MSIS‐29 have repeatedly been found (Goodwin & Green, 2015; Schäffler et al., 2013). Additionally, Rasch analysis has supported the use of two subscale scores instead of a total score (Ramp et al., 2009). A CFI of 0.90 or greater has been suggested as a criterion for an acceptable fit of the scale in a unidimensional model (Hu & Bentler, 1999). The CFA results in this study did not support the unidimensionality of the MSIS‐29 total score (CFI 0.74). The CFIs were higher for the MSIS‐29 physical and psychological subscales—0.88 and 0.89, respectively. Additionally, our finding on the differences in MSIS‐29 physical and psychological subscales according to disease severity supports the view that two separate subscale scores should be used instead of a total score. Cross‐sectional data did not allow evaluation of test‐retest reliability or responsiveness to change in MSIS‐29.

In conclusion, the Finnish version of MSIS‐29 seems to be culturally well adapted and to have sound psychometric properties, such as convergent and divergent validity and internal consistency. MSIS‐29 offers the opportunity to evaluate the physical and psychological impacts of MS rigorously from the patient's perspective.

## CONFLICTS OF INTEREST

None declared.
